# Syntenator: Multiple gene order alignments with a gene-specific scoring function

**DOI:** 10.1186/1748-7188-3-14

**Published:** 2008-11-06

**Authors:** Christian Rödelsperger, Christoph Dieterich

**Affiliations:** 1 Department of Evolutionary Biology, Max Planck Institute for Developmental Biology, Spemannstrasse 35, Tübingen, Germany; 2 Institute of Medical Genetics, Charité University Hospital, Berlin, Germany

## Abstract

**Background:**

Identification of homologous regions or conserved syntenies across genomes is one crucial step in comparative genomics. This task is usually performed by genome alignment softwares like WABA or blastz. In case of conserved syntenies, such regions are defined as conserved gene orders. On the gene order level, homologous regions can even be found between distantly related genomes, which do not align on the nucleotide sequence level.

**Results:**

We present a novel approach to identify regions of conserved synteny across multiple genomes. Syntenator represents genomes and alignments thereof as partial order graphs (POGs). These POGs are aligned by a dynamic programming approach employing a gene-specific scoring function. The scoring function reflects the level of protein sequence similarity for each possible gene pair. Our method consistently defines larger homologous regions in pairwise gene order alignments than nucleotide-level comparisons. Our method is superior to methods that work on predefined homology gene sets (as implemented in Blockfinder). Syntenator successfully reproduces 80% of the EnsEMBL man-mouse conserved syntenic blocks. The full potential of our method becomes visible by comparing remotely related genomes and multiple genomes. Gene order alignments potentially resolve up to 75% of the EnsEMBL 1:many orthology relations and 27% of the many:many orthology relations.

**Conclusion:**

We propose Syntenator as a software solution to reliably infer conserved syntenies among distantly related genomes. The software is available from .

## Background

Whole genome sequencing has boosted our knowledge database on genome architectures. Identification of conserved genomic regions across species borders has drawn much attention to the field of comparative genomics [1,2]. The identification of homologous regions between genomes supports genome annotation, function prediction and the study of evolutionary relationships between species. Depending on the level of divergence, homologous regions are usually defined by conserved orders of local genomic alignments [3], orthologous exons [4] or genes [5].

Conservation of gene order across multiple species is usually referred to as 'conserved synteny' or 'collinearity'. In the context of genome evolution, collinear blocks could be used to measure evolutionary distances between genomes in terms of genome rearrangement distances (GRD). The order of all collinear blocks in a genome can be represented as a sequence of signed integers, the GRD denotes the number of rearrangements to transform one such sequence into another [6].

The standard approach to reconstructing blocks of 'conserved synteny' is to first define a homolog assignment of gene copies. Subsequently, maximal blocks of collinearity are determined on the given homolog assignment and genomic gene orders in the compared genomes.

Traditionally, orthologs were defined by best-reciprocal BLASTP hits (BRH). For example, COGs (Cluster of Orthologous Groups, [7]) are built from cliques of size 3 in the graph of mutual best cross-species BLAST hits. These seed clusters are subsequently merged into bigger clusters provided that one side is shared between them. Other approaches (e.g. [8] or [9]) improve on this approach as they also take gene duplication and gene loss events into account.

The existence of gene families complicates homolog assignment based on protein sequence similarity. The genomic context of a gene copy might provide additional information as to the gene's evolutionary history. Gene copies that are surrounded by the same genes in different genomes are more likely to be true ancestral copies. Consequently, homolog assignment and conservation of gene orders are interlinked and should be jointly studied.

Boyer et al. [10] present a generic approach to merge information from two or more primary graphs. They explicitly discuss the problem of finding contiguous genes with conserved order across multiple genomes. Gene tuples (one gene per genome) are initially built from a set of orthology relations (protein sequence similarity and alignment coverage cutoff) and enter a multigraph as vertices. These vertices are connected by edge sets, which are defined by the gene order in the respective genomes. Subsequently, common connected components are searched that constitute blocks of conserved gene orders. The worst-case time complexity of the proposed algorithm for finding common connected components is *O*(*n*(*e*·*n *+ *m*)) where *n *is the total number of nodes in the multigraph, *e *is the number of primary graphs and *m *is the total number of edges in the multigraph. Boyer et al. [10] noticed that this procedure could be too stringent and allow the insertion of additional edges in the primary graphs. We have re-implemented this method in our Blockfinder algorithm (Additional file 3).

Conceptually more advanced approaches consider all genes of the compared genomes simultaneously. In a partial order alignment approach, a score function is used to integrate protein sequence similarity over the genomic context. Previous work on pairwise gene order alignment has been presented by Haas et al. [5] and Wang et al. [11]. Both methods resort to dynamic programming approaches that are closely related to the Smith-Waterman algorithm [12] and operate on directed acyclic graphs. Along these lines, we propose the Syntenator algorithm that facilitates multiple gene order alignments with a novel scoring function. In short, Syntenator is a hybrid approach that combines protein sequence similarity and genomic context dynamically.

### Partial gene order alignment

**Figure 1:**
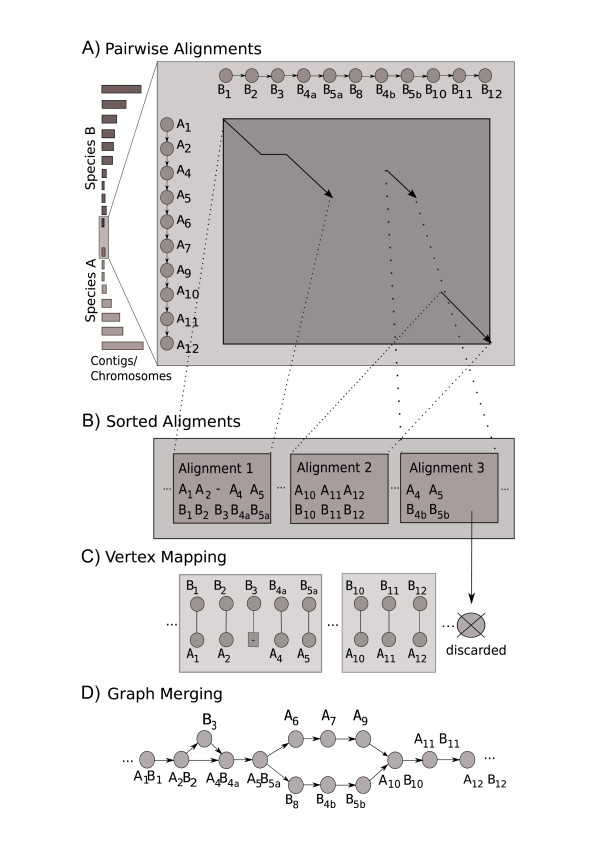
**Outline of a pairwise partial gene order alignment of two genomes**. This figure depicts all required steps to compute a pairwise gene order alignment of two genomes. Step 1 involves the pairwise comparison of all contiguous sequence regions of two species. The alignment matrix is shown for one pairwise comparison of two partially ordered gene sets: *A*_1,2,4,5,6,7,9,10,11,12 _and *B*_1,2,3,4*a*,5*a*,8,4*b*,5*b*,10,11,12_. The gene indices express homology relations (e.g. *A*_1 _is homologous to *B*_1 _and *A*_4 _is homologous to *B*_4*a *_and *B*_4*b*_). In this example three alignments were sampled from this pairwise comparison. In step 2, all alignment candidates are sorted in descending order according to their score. Alignments that do not pass a user-defined threshold are discarded. The next step (3) enforces a 1:1 mapping of "matching" vertices. Genes are greedily assigned to one another based on the sorted alignments. The final step (4) merges the two chain graphs into a partial order graph (POG). Matching nodes are "fused" and non-matching nodes are retained as individual nodes.

The Blockfinder method (as in [10]) has some important shortcomings. First, it does not use the all-against-all protein similarity search results comprehensively. Second, not all genes, just clique members are represented in the data. We get rid of these shortcomings by an approach based on partial order alignment (POA, [13]). In POA, genomes are represented as partially ordered sets. These sets contain chains (totally ordered subsets), which constitute the succession of genes on chromosomes or genomic contigs. Intuitively, these sets can be described by directed acyclic graphs. In these graphs, each node corresponds to a gene. These nodes are ordered by ascending genomic coordinates and consecutive genes are connected by directed edges (see Figure 1A).

These directed acyclic graphs are subsequently called partial order graphs (POGs). The simple instance of a POG represents a chromosome or genomic contig where each node (gene) has an in-degree = out-degree = 1 (except the start and end nodes). Two simple POGs (and DAGs in general, [14]) can be aligned by using an extension of the Smith-Waterman algorithm. Figure 1A shows the trace back matrix of a pairwise alignment of two simple POGs from species A and B. Several local alignments (above a user-defined threshold) are extracted from the trace back matrix. These alignments are ranked based on their score (see Figure 1B). This step necessitates the definition of a scoring function for genes and we will present one in the implementation section. The set of pairwise local alignments defines a gene-gene mapping or mapping of vertices of the two input graphs (Figure 1C). Three possible scenarios may occur in this simple example: two genes match, two genes do not match or one gene has no counterpart in the other graph (gap case). In the last step (Figure 1D), two POGs are merged to form a new and possibly more complex POG. All vertices of matching genes are fused into single vertices. The remaining vertices are retained as individual nodes. This merging step must yield a directed acyclic graph for the next (multiple) alignment step. This is obviously the case in our simple example but far from trivial for an alignment of two complex POGs. We will now turn to the actual implementation of Syntenator where we will discuss all relevant aspects of POG alignment.

## Implementation

### Syntenator

Syntenator combines conservation of gene order and local sequence similarity to deduce gene orthology. Partial order alignments are represented by partial order graphs (POG). We present an implementation that operates on one POG and one simple chain graph, which is a representation of a linearly ordered gene set (e.g. a genome). An extension of the concept to the alignment of two arbitrary POGs will be discussed in detail.

#### Modifications to the recurrence relation

We need to modify the recurrence relation of the traditional Smith-Waterman approach to work on POGs. To compute a maximal alignment score for a particular pairing of vertices (*n*, *m*) by dynamic programming, we need to consider all gene vertices that are linked to *n *and *m *by outgoing edges. The corresponding recurrence relation of the score function for gapped local alignments is given in Eqn 1. [14]

(1) $$S(n,m)=\underset{p\in P,q\in Q}{\max}\{\begin{array}{ll} S(p,q)+s(n,m) & \text{match/mismatch}\\ S(p,m)+\Delta & \text{insertion}\\ S(n,q)+\Delta & \text{deletion}\\ 0 & \text{start new alignment} \end{array}$$

Each cell *S*(*n*, *m*) of the dynamic programming matrix is maximized over the four possibilities: match, insertion, deletion and starting a new alignment. The main difference to traditional pairwise local alignment are *P *and *Q*, the sets of predecessor nodes of *n *and *m *in the corresponding POGs. For complex POGs, we have to consider |*P*| × |*Q*| alternative candidates in case of a match. The most simple case is |*P*| = |*Q*| = 1 if we were to align two genomes. Our implementation operates on one POG and one simple chain. Consequently, we have either |*P*| = 1 or |*Q*| = 1. The expressions *s*(*n*, *m*) and Δ denote the match score for two nodes and the gap penalty, respectively.

#### Gene order alignment

Initially, all pairwise alignments between two POGs (e.g. *G*_1 _and *G*_2_) are computed in forward and reverse direction. An alignment in the reverse direction requires the reversal of all edges in one of the two POGs. For each comparison (in both directions), we consider all local (sub)optimal alignments above a certain threshold Θ. All alignments are ranked by their scores in descending order. Based on these alignments, we decide which vertices match and should be fused into a common vertex. We greedily assign vertex matches by traversing the ordered list top-down.

Algorithm 1 (see Appendix) shows the adaptations of the algorithm of Lee et al. [13] to produce a set of all suboptimal alignment paths *P*. Such a path consists of a tuple (*s*, *L*, *r*) where *s *denotes the score, *L *is a list of aligned node pairs and *r *indicates wether a gene order was aligned in its original or reversed orientation. The score is adjusted by subtracting the initial score *s*_*init *_which is defined as the last minimal score encountered during traceback before the score exceeds the final alignment score or 0 if no such minimum exists. This adjustment is necessary to prevent that alignments inherit scores from previous higher scoring alignments.

#### Merging genome graphs

In POA, two graphs, *G*_1 _and *G*_2_, are merged after each round of pairwise alignments. We have already discussed how to identify pairs of vertices (e.g. (*v*, *w*) with *v *∈ *G*_1 _and *w *∈ *G*_2_) that should be merged between both graphs. We denote this as 1:1 mapping *M*.

In the merging step, we iterate over all vertices *w *∈ *G*_2 _and add a copy of *w *to *G*_1 _if *w *∉ *M*. If (*v*, *w*) ∈ *M *we fuse *v *and *w *by copying the genes stored at *w *to *v*. If a *G*_1_-equivalent of the predecessor node of *w *exists, we connect this *G*_1_-equivalent predecessor node of *w *to *v*. All connections between nodes that were not fused, but simply added to the graph, are retained in the merged graph.

**Figure 2:**
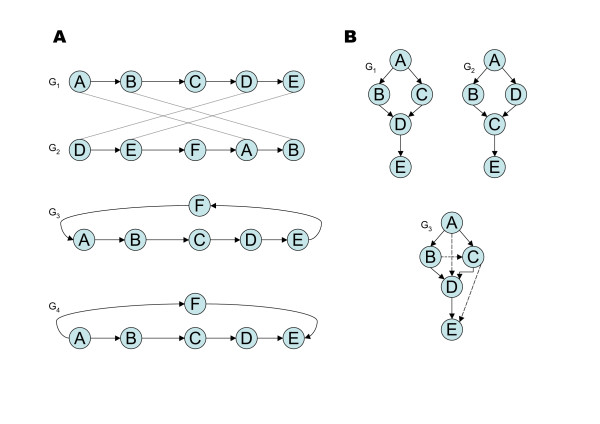
**Removing cycles after merging POGs**. Panel A depicts the situation where two local gene order alignments "cross". Matches between nodes are shown as dashed connections between *G*_1 _and *G*_2_. *G*_3 _shows the situation after the merging step where a loop has introduced a cycle. This cycle is detected by the program and removed by reversing all edges (see text). The final POG looks like *G*_4_. Panel B depicts the scenario where two local alignments exist in different orientations (A-B in *G*_1_, *G*_2 _and C-D in *G*_1_, *G*_2_*r*). *G*_3 _shows the final POG after merging and cycle removal. Solid edges stem from the reference graph *G*_1_. The two dashed edges have been introduced to represent order relations that are unique to *G*_2_. The edge from D to C in *G*_2 _would introduce a cycle and had to be removed. The "kinked" edge represents the alignment of C→D in *G*_1 _to D→C in *G*_2_.

The merging of two POGs may introduce cycles into the resulting POG for two reasons: 1) Local alignments are not collinear in the respective input POGs (Figure 2A). 2) Local alignments are produced in both orientations (forward and reverse, Figure 2B).

These particular problems did not arise in the original implementation for protein or EST sequence alignment (e.g. [14]) where DAGs are aligned in one defined orientation (e.g. N to C terminus for proteins, 5' to 3' end for ESTs) and just one optimal alignment is reported.

To resolve newly introduced cycles in scenario 1 (Figure 2A), we use a topological ordering of *G*_1 _and check at all branching points, whether a loop path consisting of new nodes from *G*_2 _induces a cycle in the merged graph *G*_3_. We have to test if the loop path returns to a node in *G*_1 _at an index which is less or greater in terms of the topological order than the index of the branching point from which we started off. If the path is a forward path and the index of the returning point is smaller than the index of the branching point, all edges within the path have to be reversed to keep the graph acyclic. This procedure leads to *G*_4 _in Figure 2A. The case for the backward path works analogously. If the newly added loop is part of a greater loop in *G*_1_, we have to search in both directions for the endpoints of the old loop to define an order relation on the newly added loop.

The second case (Figure 2B) emerges if local alignments of opposite orientations exist. In the given example, a cycle would be formed between nodes C and D as they are aligned in opposite orientation to A and B. This is circumvented by keeping the edge orientation of one graph (*G*_1_) for the reverse alignment. The "dashed" edges are added to preserve the original order relations of *G*_2_.

Repetitive regions that may result from duplication events do not introduce cycles into the merged POG since we greedily enforce a 1:1 mapping of gene nodes. Only the best matching repeat copies would be merged.

#### Score function

Our algorithm relies on BLASTP hits as general similarity measure. From the set of all-against-all BLASTP hits, we save a bitscore for each gene pair in a lookup table. In case of alternative transcripts the highest score between any two protein products is saved.

We chose a scoring function that allows us to order alignments according to the number of aligned pairs or to the sum of pairwise similarities in case of equal numbers of pairs.

For each pair of genes (*A*, *B*) a symmetric score function is given by Eqn. 2. The individual contributions are shown in Eqn. 3.

(2) *S*_*match *_(*A*, *B*) = *s*(*A*, *B*) + *s*(*B*, *A*)

(3) $$s(A,B)=1-\frac{1}{s_{\text{bitscore}}(A,B)}$$

We require *s*_bitscore _to be ≥ 50. The match score is always < 2: $\underset{s_{\text{bitscore}}\to \infty }{\lim}S_{match}(A,B)\to 2$.

This can be interpreted as summing up over the entries of a non-symmetric weighted adjacency matrix of all pairwise homology relationships. A mismatch score is assigned if the two genes under comparison either have no BLAST hit or if they are located on different strands.

In order to score a match of vertices which contain multiple genes, we use a normalized sum-of-pairs score (Eqn. 4).

(4) $$S(v,w)=\frac{\sum_{A\neq B;\;A,B\in \text{geneset}(v\cup w)}S(A,B)}{\frac{C_{v,w}(C_{v,w}-1)}{2}}$$

(5) $$C_{v,w}=\{\begin{array}{ll} n_{v,w} & \text{if }\sigma <\Theta \\ n(G_{v},G_{w}) & \text{else} \end{array}$$

*n*_*v*, *w *_denotes the number of genes of nodes *v *and *w*, $n_{\left(G_{v},G_{w}\right)}$ denotes the number of species in the graphs of *v *and *w*. The term *C*_*v*, *w *_in the denominator of Eqn. 4 is a scaling factor whose definition depends on the current alignment score. *C*_*v*, *w *_is equal to the number of comparisons between either all species in nodes *v *and *w *or the number of all species in the graphs of *v *and *w *(Eqn. 5). This correction scheme was implemented because weak BLAST hits tend to appear in the set of genes of both vertices more often if the number of compared genes increases. As a consequence pairwise scores tend to be higher than the averaged scores of multiple comparisons. In order to equalize this effect, we replace *n*_*v*, *w *_by $n_{\left(G_{v},G_{w}\right)}$ as soon as the alignment score *σ *exceeds the threshold Θ. This triggers a switch towards a more specific search for alignments containing genes from multiple species.

## Results

We applied both approaches to detect conserved syntenies in four mammalian species, namely human (NCBI 36), mouse (NCBI m36), rat (RGSC 3.4) and dog (CanFam 1.0). We computed all pairwise all-against-all BLASTP searches in advance. The BLASTP hit ranks and bitscores are subsequently used by Blockfinder and Syntenator. The number of genes with putative homologs at an E-value cutoff < 0.1 is shown in Additional File 1. Only these genes are considered in whole genome alignments. We contrasted our findings to the EnsEMBL compara database, which reports pairwise conserved synteny relations based on nucleotide alignments.

### Application of Syntenator

We used the aforementioned data to construct POGs for all genomes. Classical methods like best reciprocal hits and COGs [15] select best BLAST hits to assign orthologs. We suggest that in order to maximize conserved synteny, non-best hits should be taken into account. Nevertheless highly abundant protein domains drastically increase the number of BLAST homologies for certain genes [9,15] but these homologs are unlikely to be true 1:1 orthologs. In order to reduce the amount of data being passed on to Syntenator we apply certain filters: The BLAST similarity relations were filtered to contain only the 5 best hits per query. Hits were further removed if their bitscore dropped below 95% of the best score.

If we chose to include more BLAST similarity relations per gene, more alignments would pass the minimal threshold Θ. That is why, the actual choice of the BLAST similarity relations is a tradeoff between speed and sensitivity. Our filtering step cuts down on the number of candidate alignments that would have to be evaluated.

Syntenator was run on this data set using a linear gap score of *-*2.0, a mismatch score of *-*3.0 and a threshold of 2.0. These values were motivated by assuming that a complete loss of two genes is less likely than a mismatch between two diverging genes. A threshold of 2.0 requires that a pairwise ungapped alignment consists of at least two gene pairs.

#### Pairwise genome comparison

We compared the performance of gene order alignment approaches (Blockfinder and Syntenator) to the EnsEMBL compara synteny data set. Herein, Blockfinder utilized three homology data sets, which are all based on EnsEMBL release 46: 1) Ensembl orthologs (1:1, 1:many and many:many), 2) Best reciprocal BLASTP hits (BRH) and 3) 3-best-reciprocal BLASTP hits (BRH3). In the last case, a gene may have up to 3 hits. Generally, a gene node may have up to (*g *- 1) * *n *homology relations to other genes, where *g *is the number of species and *n *is the number of considered BLASTP hits.

We first consider the well studied man-mouse species pair, which is separated by a small phylogenetic distance. A previous study [16] reported that ~40% of the two genomes align on the nucleotide level. A comparison of Blockfinder, Syntenator and nucleotide level alignments tells us two things:

1. How much conserved synteny information we lose as compared to the "gold" standard as given by the EnsEMBL compara data

2. How much we improve over simpler methods that define homology relations in advance (e.g. Blockfinder).

**Figure 3:**
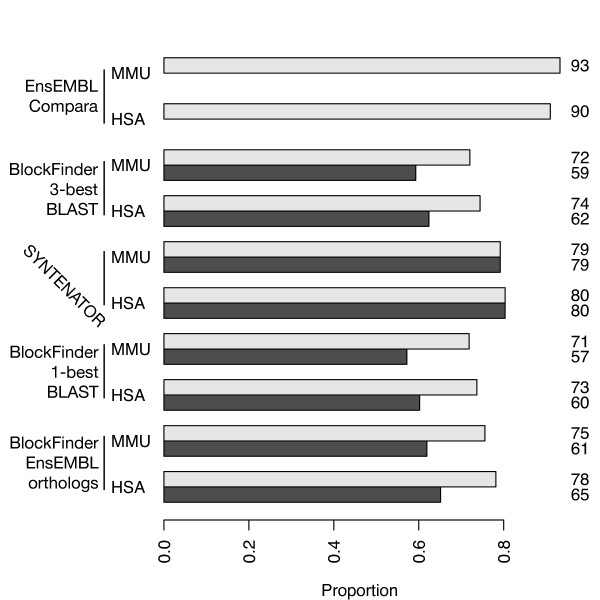
**Pairwise comparison of the human and mouse genome with Blockfinder and Syntenator**. Dark gray bars represent the proportion of man (HSA) and mouse (MMU) genes, which could be aligned. Light gray bars represent the proportion of human and mouse genes, which fall into regions that are covered by alignments. Both number are the same for Syntenator as it considers all genes of a genome simultaneously. The "EnsEMBL Compara" bars are taken from the EnsEMBL synteny blocks. The three other runs were conducted with BlockFinder and differing sets of homology relations (see text). The number to the right of each bar is the proportion relative to the total gene set in percentage.

Figure 3 shows a comparison of these methods for a pairwise whole genome alignment of man and mouse. Syntenator aligned more human genes than Blockfinder (dark gray bars). Furthermore, Blockfinder covered less genes with conserved segments than Syntenator (80% versus maximally 78%, light gray bars). In other words, Syntenator shows the highest genome coverage after the EnsEMBL compara synteny data set. Considering the intersection of the two data sets, we noticed that Syntenator overlaps with 80% of the conserved syntenic EnsEMBL compara regions (93% for the reverse comparison). The reason why we miss out 20% of the Compara set is quite simple. The Compara data set is generated from "chained" collinear nucleotide level alignments. Consequently, conserved syntenic regions are not necessarily completely covered by nucleotide level alignments. This is the main reason why the Compara data set covers more genes. Additionally, our parameter setting is rather conservative with the effect that alignments might terminate too early. Nevertheless, we could clearly demonstrate that our parameter setting was sufficient to outperform solutions which define orthology relationships prior to alignment.

However, the full potential of our method unfolds when two remotely related species are aligned. We compared whole genome alignment coverage of Syntenator and UCSC blastz runs on the nucleotide level. Blastz [16] is a pairwise whole genome alignment method, which produces local nucleotide sequence alignments.

**Figure 4:**
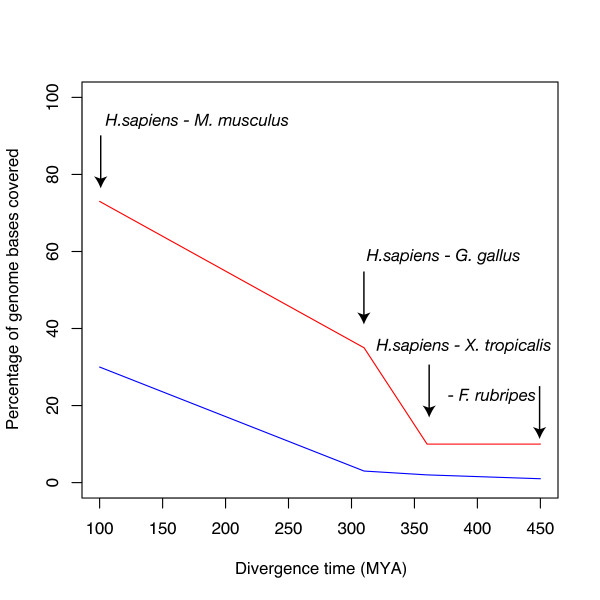
**Comparison of whole genome coverage for Syntenator and UCSC blastz alignments**. This figure shows the proportion (in %) of the human genome that is covered by Syntenator (red line) or UCSC blastz (blue) alignments. We performed four pairwise genome comparisons with increasing evolutionary distance (see labels). The estimated divergence times are shown on the x-axis.

Figure 4 shows the proportion of the human genome basepairs that are covered by either gene order alignments (red line) or nucleotide level alignments (blue line). We calculated this proportion by summing up all bases that fall into alignment regions as defined by alignment start and end coordinates. Genome coverage of nucleotide level alignments shrinks dramatically with increasing evolutionary distance. Gene order alignments generally cover a greater proportion of the genome than nucleotide level alignments do. The biggest difference is seen for the human-chicken comparison where 35% of the human genome is covered by gene order alignments as opposed to a coverage of 3% for nucleotide sequence alignments.

#### Multiple genome comparison

Syntenator was also used to compute multiple gene order alignments between man, mouse, rat and dog. The species were aligned progressively in two different orders: Human, dog, mouse and rat (HDMR) and Mouse, rat, human and dog (MRHD). Alignment parameters were changed to a mismatch score of -8 and a gap penalty of -3. This choice of parameters penalizes genes that match only to a subset of genes of a POG node more effectively in our sum-of-pairs score setting. Table 1 summarizes the two four-genome alignments. We observed that up to 78% of the primary genomes end up in Syntenator blocks.

**Table 1:** Syntenator multiple gene order alignments

**Alignment order: MRHD**	Mouse	Rat	Human	Dog
Number of blocks	304	301	289	328

Mean length	48.2	41.4	44.8	42.7

Genes in alignments	14665 (60%)	12451 (53%)	12933 (56%)	14018 (77%)

Block size in Mb	1820.1	1832.0	2416.6	1694.4


**Alignment order: HDMR**	Mouse	Rat	Human	Dog

Number of blocks	367	397	345	331

Mean length	42.6	45.7	42.6	36.3

Genes in alignments	15650 (64%)	18151 (78%)	14698 (63%)	12036 (66%)

Block size in Mb	1772.6	1923.6	2438.4	1815.4

Two alternative Syntenator multiple gene order alignments of mouse (M), rat (R), human (H) and dog (D) are shown to demonstrate the dependence of the alignment outcome on the alignment order of species. The proportion of genes in alignments relative to the total gene set is shown in percentage. The mean length of a block is the ratio of Genes in alignments/Number of blocks.

In the last round of the multiple alignment, either the POG of human, dog and mouse is aligned to the rat genome or the POG of mouse, rat and human is aligned to the dog genome. It is apparent that the final multiple gene order alignment is sensitive to the order of alignment steps. After this last alignment round, Syntenator reports only genes that have been aligned with the genome that was added last (either dog or rat). This is also reflected in Table 1 where the genome that was added last shows the highest percentage of aligned genes. Please note that multiple genome alignments do not necessarily contain genes from all species. In total, there are 11,164 and 11,309 alignment nodes in the MRHD and HDMR POGs that consist of 4-tuples (nodes with one gene from each species). This is close to the lowest number of genes from a single species in the two multiple gene order alignments (see Table 1). Future work will address alternative scoring schemes as well as a more rigorous assessment of the impact of alignment orders.

### Comparison of orthology prediction

Another potential application of Syntenator is its use to assign gene homology relations. To this end, we compared orthology predictions of Syntenator and the EnsEMBL system. Whole genome alignments with Syntenator were performed with an alignment score threshold of 1.0 so that a prediction is made for each gene with a homolog. The other parameters were set to -3 for a mismatch and -2 for a gap. Firstly, we checked how many EnsEMBL 1:1 orthologs are contained in the Syntenator gene pairs. Except for the man/mouse comparison (94%), ~97% of all EnsEMBL 1:1 orthologs are also predicted by Syntenator (see Additional File 2). Many instances of 1:many or many:many EnsEMBL orthologs could be formally resolved to 1:1 orthologs (see Additional File 1). Resolving 1:many and many:many relations bears the question of what a true ortholog is. We argue for defining orthology on protein sequence similarity and gene order. This argument has been previously made in the context of gene function prediction [17]. Our Syntenator framework accomplishes this task. However, a good test set is not available to our knowledge and simulating whole genome evolution is beyond the scope of this manuscript.

## Conclusion

We have established Syntenator as a new method to identify regions of gene order conservation over multiple genomes. Furthermore, we propose that our method could be used to resolve gene homologies. Instead of defining an orthologous group from sequence similarity alone, our method chooses the ortholog from a set of candidate genes according to available synteny information. This observation is necessary as relying on best reciprocal hits exclusively does not guarantee to find the 'true' ortholog. This circumstance might be explained by the weakened selective pressure on duplicated genes [18].

Blockfinder chooses orthologs from a set of candidate orthologous genes by maximizing collinearity across all species. The initial clique graph does not capture all existing BLAST homologies. Genes outside of cliques are excluded from the subsequent analysis. In general, this is a disadvantage but turns into an advantage when genomes with poor gene annotations are used.

Syntenator integrates all gene positions and complete BLAST data into the computation of collinear blocks. Herein, synteny information is used as the first criterion to define orthology, although substantial local sequence similarity as expressed by BLAST scores is still required.

In summary, our work extends existing methods for orthology prediction and provides new tools to compare local and global genome architectures of multiple species, especially for genomes that do not align on the nucleotide level.

## Availability and requirements

**Project name: **Syntenator

**Project home page: **

**Operating system(s): **Platform independent

**Programming language: **Java

**Other requirements: **Java 1.4.2 or higher

**License: **freely available to academia

**Any restrictions to use by non-academics: **license is needed

## Competing interests

The authors declare that they have no competing interests.

## Authors' contributions

CD conceived the project and provided conceptional support to CR. CR implemented the algorithms and carried out all data analyses. CD wrote the manuscript.

## Appendix

### Algorithm 1: Computing a set of suboptimal gene order alignments

*N*, *M *are the number of nodes in both graphs. *A *is the dynamic programming matrix and *T *is the traceback matrix. Cells of *T *contain the index tuple of the predecessor cell pointing to any cell in the computed area. The indices *i *and *j *iterate over the topological orders of both graphs (line 5,6).

1: **for ***i *← 0 **to ***N ***do**   // initialize matrices

2:   *A*(0,*i*) ← 0, *T*(0,*i*) ← (0, 0)

3: **for ***j *← 0 **to ***M ***do**

4:   *A*(*j*, 0) ← 0, *T*(*j*, 0) ← (0, 0)

5: **for ***i *← 1 **to ***N ***do**   // dynamic programming

6:   **for ***j *← 1 to *M ***do**

7:      (*A*(*j*, *i*),*T*(*j*, *i*)) ← Score(*j*, *i*, *A*, *T*)

8:      (*p*_*j*_,*p*_*i*_) ← *T *(*j*, *i*)

9:      **if ***A*(*j*, *i*) > *A*(*p*_*j*_,*p*_*i*_) **then**

10:         *L *← *L *∪ (*A*(*j*, *i*),*j*, *i*) // store each cell with increasing score

Score(*j*, *i*, *A*, *T*) fills cells *A*(*j*, *i*) and *T*(*j*, *i*) according to Section "Score function" and Eqn. 1 (line 7).

Subsequently the scores *A*(*j*, *i*) and *A*(*p*_*j*_,*p*_*i*_) are compared and cells with increasing score are stored as candidates in *L *(line 9,10). The candidate alignments in *L *are processed by decreasing score. An alignment path *p *is stored, if the difference *s - s*_*init *_exceeds the threshold Θ (line 14–16).

11: **for ***k *← **to **|*L*| **do**

12:   (*s*, *j*, *i*) ← *L*(*k*)

13:   *s*_*init *_← InitialScore(*j*, *i*, *A*, *T*)

14:   **if ***s - s*_*init *_>*Θ ***then**

15:      *p *← Traceback(*j*, *i*, *s*_*init*_,*A*, *T*)

16:      *P *← *P *∪ *p*   // update the sorted set of paths

## Supplementary Material

Additional file 3 **Brief description of the Blockfinder implementation**. This text document overviews our Blockfinder implementation, which we used as a reference implementation of a generic approach to merge information from two or more primary graphs. Click here for file

Additional file 1 **Resolving EnsEMBL 1:many and many:many orthologs**. This table shows how many ambiguous pairwise orthology relations as defined by the EnsEMBL pipeline could be resolved with Syntentator. Click here for file

Additional file 2 **Supplementary Tables**. Table 1 – BLASTP Homologs. Amount of genes for which BLASTP homologs could be detected (E-value < 0.1). Table 2 – One-one orthologs. Amount of Ensembl40 one-to-one orthologs which were recoverved by Syntenator. Table 3 – Overlap between syntenic blocks. For each pairwise comparison and each method, the ratio between sequence in overlapping blocks and total sequence in blocks is shown. Table 4 – Comparison of conserved synteny predictions for genome pairs. BF stands for Blockfinder. BRH denotes Best reciprocal BLAST hits. Genome data and compara data were retrieved from the EnsEMBL database release 40. Click here for file
