# Supplementary material for: Syntenator: Multiple gene order alignments with a gene-specific scoring function
Source: Algorithms Mol Biol. 2008 Nov 6;3:14. doi: 10.1186/1748-7188-3-14 (PMC2590594; doi:10.1186/1748-7188-3-14)
Supplement: Additional file 3 — Brief description of the Blockfinder implementation. This text document overviews our Blockfinder implementation, which we used as a reference implementation of a generic approach to merge information from two or more primary graphs. [file 1748-7188-3-14-S3.pdf]

BLOCKFINDER

The program BLOCKFINDER takes a set of pairwise cross-species protein similarity relations as input. The program defines cliques of orthologous genes and returns conserved paths through the resulting clique order graph as output.

Cliques of orthologous genes

A set of all-against-all BLASTP comparisons is generated in a preprocessing step. Subsequently, a homology graph is constructed out of the set of n-best-reciprocal BLAST hits where n is the number of BLAST hits that are considered per gene. Genes are represented as nodes and edges correspond to homology relations. A gene pair get connected if both genes are among the n-best targets in a reciprocal BLAST search. In other words, a gene node may have up to $(g-1)*n$ homology relations to other genes, where g is the number of species.

To identify the orthologous groups we split this graph into its connected components and scan each component for cliques of maximum size, that is subsets of vertices in which an edge exists for each pair of vertices in the set. Clique searching is an NP-complete problem (1), but in our case the maximum clique size k is small enough that we can use a simple algorithm. We choose $k = 4$ for our evaluation example. All connected pairs of proteins from different species serve as initial cliques. Cliques are augmented by looking for a vertex which is connected to all vertices in the current clique. This is repeated until a maximal clique size is reached.

We decided to use n-best-reciprocal BLAST hits instead of BRHs in order to choose the ortholog from a greater set of candidate genes. Evidently, this comes at a high price. The size of components increases drastically with the number of non-best hits, a phenomenon known as ”sprawling” (2). As a consequence, n should be small. We computed examples for the top three BLAST hits ($n = 3$).

Identification of collinear blocks

By mapping the cliques onto the genomic loci of the genes, each chromosome or contig can be represented by a signed clique permutation. This can be transformed into a directed acyclic graph (DAG) with vertices corresponding to cliques and edges between nodes v, w if the gene of the clique in v precedes the gene associated with w in the respective genome.

Collinear blocks are now defined as paths of conserved clique orders. These can be extracted in two steps. First the graphs of all species are merged at vertices representing the same cliques. We introduce an edge multiplicity, which we denote m. m represents the number of species which support this clique order. New cliques are added as additional vertices and edges are either added or their multiplicity is incremented. Algorithm 1 shows how two genome graphs G_1 and G_2 are merged. Due to arbitrary strand labeling between assemblies, we try to match a candidate edge in forward and reverse direction (after changing order and orientation). The resulting graph stores all order relationships between all cliques in all species.

In the second step all longest paths of edges with $m > 1$ are searched via depth first search (3). The constraint $m > 1$ ensures that the corresponding gene pair exists in at least two genomes. Found paths correspond to collinear blocks. Each vertex is assigned to one path only. Another program parameter d sets an upper bound on allowed gap sizes. Every clique member is checked for being less than d genes away on the genome level from the corresponding gene of the preceding clique. We set $d = 2$. This eliminates the possibility of identifying regions of conserved synteny where a small number of genes are scattered throughout the compared chromosomes. The minimal number of cliques is set to 3. The smallest possible block is then given by three consecutive gene cliques.

Algorithm 1: Clique order graph construction.

G_1 represents the clique order graph of possibly multiple species. The following pseudocode shows how a new species, represented by G_2 can be attached to G_1. For each edge in E_2, either a new edge in G_1 is created (line 16) or the multiplicity of an existing edge is incremented (line 10,14).

1: $G_1 := (V_1, E_1)$, $G_2 := (V_2, E_2)$
2: **for** $i \leftarrow 0$ **to** $|E_2|$ **do**
3: $e_2 \leftarrow E(i)$
4: $v_2 \leftarrow$source(e_2)
// source(e) returns the source node for a directed edge e
5: $w_2 \leftarrow$target(e_2) // target(e) analogously
6: $v_1 \leftarrow$getNode(G_1, v_2)
7: $w_1 \leftarrow$getNode(G_1, w_2)
// v_1,w_1 are the equivalent vertices of v_2,w_2 in G_1

getNode(G, v) returns a node in G corresponding to v. If no such node exists, a new node is created in G and is returned.

8: $e_1 \leftarrow (v_1, w_1)$
9: **if** $(e_1 \in E_1 \wedge$ ori(v_1) = ori$(v_2) \wedge$ ori(w_1) = ori$(w_2))$ **then**
10: $m(e_1) \leftarrow m(e_1) + 1$
// increment multiplicity of e if both orientations are equal
11: **else**
12: $e_r \leftarrow (w_1, v_1)$ // test reversed case
13: **if** $(e_r \in E_1 \wedge$ ori$(v_1) \neq$ ori$(v_2) \wedge$ ori$(w_1) \neq$ ori$(w_2))$ **then**
14: $m(e_r) \leftarrow m(e_r) + 1$
15: **else**
16: $E_1 \leftarrow E_1 \cup e_1$ // add e to G_1
17: $m(e_1) \leftarrow 1$

References

[1] Karp RM: *Complexity of Computer Computations*, Plenum 1972 chap. Reducibility Among Combinatorial Problems, :85–103.

[2] Goodstadt L, Ponting CP: **Phylogenetic reconstruction of orthology, paralogy, and conserved synteny for dog and human.** *PLoS Comput Biol* 2006, **2**(9):e133, [http://dx.doi.org/10.1371/journal.pcbi.0020133].

[3] Cormen TH, Leiserson CE, Rivest RL, Stein C: *Introduction to Algorithms*. The MIT Press; 2 edition 2001.
