# Supplementary material for: Syntenator: Multiple gene order alignments with a gene-specific scoring function
Source: Algorithms Mol Biol. 2008 Nov 6;3:14. doi: 10.1186/1748-7188-3-14 (PMC2590594; doi:10.1186/1748-7188-3-14)
Supplement: Additional file 1 — Resolving EnsEMBL 1:many and many:many orthologs. This table shows how many ambiguous pairwise orthology relations as defined by the EnsEMBL pipeline could be resolved with Syntentator. [file 1748-7188-3-14-S1.pdf]

Table 2 - Resolving EnsEMBL 1:many and many:many orthologs

EnsEMBL1:many orthologs		Genome A			Genome B			best hit heuristic
Genome A	Genome B	1:many	resolved	local duplication	1:many	resolved	local duplication	resolved
Human	Mouse	543	359 (67%)	110 (20%)	307	200 (65%)	49 (16%)	77%
Human	Rat	552	374 (68%)	88 (16%)	301	189 (63%)	45 (15%)	77%
Human	Dog	210	157 (75%)	17 (8%)	364	231 (64%)	71 (20%)	77%
Mouse	Rat	505	345 (68%)	80 (16%)	538	332 (62%)	107 (20%)	77%
Mouse	Dog	171	126 (74%)	16 (9%)	562	371 (66%)	104 (19%)	78%
Rat	Dog	573	395 (69%)	88 (15%)	174	122 (70%)	15 (9%)	81%
EnsEMBL many:many orthologs		Genome A			Genome B			best hit heuristic
Genome A	Genome B	many:many	resolved	local duplication	many:many	resolved	local duplication	resolved
Human	Mouse	471	102 (22%)	144 (30%)	586	96 (16%)	138 (22%)	54%
Human	Rat	422	88 (21%)	119 (28%)	569	83 (15%)	112 (20%)	54%
Human	Dog	203	55 (27%)	30 (15%)	194	47 (24%)	33 (17%)	59%
Mouse	Rat	828	181 (22%)	176 (21%)	769	161 (21%)	187 (24%)	49%
Mouse	Dog	217	53 (24%)	36 (12%)	184	51 (28%)	37 (20%)	53%
Rat	Dog	428	46 (11%)	29 (7%)	173	40 (23%)	35 (20%)	62%

This table shows ambiguous pairwise orthology relations as defined by EnsEMBL. The first two columns denote the compared genomes. The third column lists the total number of 1:many or many:many relations for the respective genome. Columns labeled with "resolved" show the number of ambiguous relations that could be assigned to a gene pair. Columns labeled with "local duplications" show the number of ambiguous relations due to local gene duplications. We could identify local gene duplications but did not resolve them as a resolution would not be supported by the genomic context. The column for the best-hit heuristic gives the proportion of SYNTENATOR resolutions in percentage that are also found by just taking the best BLAST hit of Genome A to Genome B to resolve ambiguities.
