# Supplementary material for: Syntenator: Multiple gene order alignments with a gene-specific scoring function
Source: Algorithms Mol Biol. 2008 Nov 6;3:14. doi: 10.1186/1748-7188-3-14 (PMC2590594; doi:10.1186/1748-7188-3-14)
Supplement: Additional file 2 — Supplementary Tables. Table 1 – BLASTP Homologs. Amount of genes for which BLASTP homologs could be detected (E-value < 0.1). Table 2 – One-one orthologs. Amount of Ensembl40 one-to-one orthologs which were recoverved by Syntenator. Table 3 – Overlap between syntenic blocks. For each pairwise comparison and each method, the ratio between sequence in overlapping blocks and total sequence in blocks is shown. Table 4 – Comparison of conserved synteny predictions for genome pairs. BF stands for Blockfinder. BRH denotes Best reciprocal BLAST hits. Genome data and compara data were retrieved from the EnsEMBL database release 40. [file 1748-7188-3-14-S2.pdf]

	Genes	Human	Mouse	Rat	Dog
Human	23224	-	20492 (88%)	19961 (86%)	20091 (87%)
Mouse	24438	21460 (88%)	-	22150 (91%)	21121 (87%)
Rat	23299	19727 (85%)	21854 (94%)	-	21809 (94%)
Dog	18214	17657 (97%)	17265 (95%)	17705 (97%)	-

Table 1: **BLASTP Homologs.** Amount of genes for which BLASTP homologs with Evalue < 0.1 could be detected.

one2one	Ensembl	Syntenator	Overlap
Human Mouse	13093	16501	12278 (94%)
Human Rat	12485	16955	12072 (97%)
Human Dog	13140	16382	12783 (97%)
Mouse Rat	15765	19125	15235 (97%)
Mouse Dog	12448	15845	12023 (97%)
Rat Dog	11891	15198	11467 (96%)

Table 2: **One-one orthologs.** Amount of Ensembl40 one-to-one orthologs which were recoverved by SYNTENATOR.

**Human Mouse**	BF on BRH	BF on EnsEMBL orthologs	SYNTENATOR	BF on 3-BRH	EnsEMBL compara
BF on BRH	1.00	0.79	0.88	0.87	0.92
BF on EnsEMBL orthologs	0.85	1.00	0.88	0.79	0.90
SYNTENATOR	0.80	0.74	1.00	0.74	0.93
BF on 3-BRH	0.94	0.79	0.89	1.00	0.90
EnsEMBL compara	0.72	0.65	0.80	0.65	1.00

**Human Rat**	BF on BRH	BF on EnsEMBL orthologs	SYNTENATOR	BF on 3-BRH	EnsEMBL compara
BF on BRH	1.00	0.90	0.84	0.88	0.92
BF on EnsEMBL orthologs	0.80	1.00	0.83	0.72	0.91
SYNTENATOR	0.74	0.83	1.00	0.68	0.93
BF on 3-BRH	0.95	0.88	0.83	1.00	0.90
EnsEMBL compara	0.64	0.71	0.72	0.57	1.00

**Human Dog**	BF on BRH	BF on EnsEMBL orthologs	SYNTENATOR	BF on 3-BRH	EnsEMBL compara
BF on BRH	1.00	0.92	0.94	0.88	0.84
BF on EnsEMBL orthologs	0.89	1.00	0.93	0.81	0.85
SYNTENATOR	0.84	0.86	1.00	0.76	0.86
BF on 3-BRH	0.96	0.91	0.93	1.00	0.82
EnsEMBL compara	0.80	0.84	0.91	0.71	1.00

**Mouse Rat**	BF on BRH	BF on EnsEMBL orthologs	SYNTENATOR	BF on 3-BRH	EnsEMBL compara
BF on BRH	1.00	0.88	0.91	0.84	0.88
BF on EnsEMBL orthologs	0.89	1.00	0.93	0.78	0.89
SYNTENATOR	0.78	0.78	1.00	0.68	0.88
BF on 3-BRH	0.96	0.88	0.91	1.00	0.86
EnsEMBL compara	0.68	0.68	0.79	0.58	1.00

**Mouse Dog**	BF on BRH	BF on EnsEMBL orthologs	SYNTENATOR	BF on 3-BRH	EnsEMBL compara
BF on BRH	1.00	0.92	0.87	0.84	0.96
BF on EnsEMBL orthologs	0.86	1.00	0.88	0.76	0.96
SYNTENATOR	0.80	0.85	1.00	0.71	0.97
BF on 3-BRH	0.94	0.90	0.87	1.00	0.93
EnsEMBL compara	0.73	0.78	0.81	0.63	1.00

Table 3: **Overlap between syntenic blocks.** For each pairwise comparison and each method, the ratio between sequence in overlapping blocks and total sequence in blocks is shown. For example for human and mouse, 93% of the blocks predicted by SYNTENATOR, overlap with EnsEMBL compara blocks. but only 80% of compara blocks overlap with SYNTENATOR.

Method	BF on EnsEMBL orthologs		BF on BRH		SYNTENATOR		BF on 3-BRH		EnsEMBL compara	
	human	mouse	human	mouse	human	mouse	human	mouse	human	mouse
Number of blocks	1447	1447	954	954	510	510	1275	1275	340	331
Mean length	12.55	12.77	17.94	18.41	36.58	37.95	13.56	13.81	62.14	68.86
Genes in segment	18153	18473	17112	17562	18658	19355	17289	17606	21129	22792
Genes in blocks	15129	15129	13981	13981	18658	19355	14491	14491	n.d.	n.d.
Block size	2012.1 Mb	1719.9 Mb	2055.6 Mb	1783.4 Mb	2245.2 Mb	1948.6 Mb	1955.6 Mb	1682.5 Mb	2644.3 Mb	2395.6 Mb

Method	BF on EnsEMBL orthologs		BF on BRH		SYNTENATOR		BF on 3-BRH		EnsEMBL compara	
	human	rat	human	rat	human	rat	human	rat	human	rat
Number of blocks	1032	1032	895	895	565	560	1160	1160	289	296
Mean length	16.06	16.13	16.65	16.78	31.22	31.49	12.97	12.95	66.80	67.42
Genes in segment	16575	16644	14903	15021	17638	17635	15042	15019	19306	19955
Genes in blocks	12862	12862	11993	11993	17638	17635	12376	12376	n.d.	n.d.
Block size	1959.0 Mb	1773.2 Mb	1734.5 Mb	1579.7 Mb	1953.8 Mb	1766.8 Mb	1669.4 Mb	1510.7 Mb	2442.0 Mb	2352.6 Mb

Method	BF on EnsEMBL orthologs		BF on BRH		SYNTENATOR		BF on 3-BRH		EnsEMBL compara	
	human	dog	human	dog	human	dog	human	dog	human	dog
Number of blocks	902	902	874	874	322	321	1117	1117	218	215
Mean length	19.32	16.97	19.32	16.95	59.82	51.92	15.18	13.33	86.71	72.31
Genes in segment	17429	15308	16890	14814	19262	16666	16960	14889	18902	15546
Genes in blocks	13284	13284	13157	13157	19262	16666	13561	13561	n.d.	n.d.
Block size	2284.3 Mb	1912.4 Mb	2214.0 Mb	1855.6 Mb	2464.9 Mb	2064.6 Mb	2107.4 Mb	1765.4 Mb	2316.8 Mb	1932.4 Mb

Method	BF on EnsEMBL orthologs		BF on BRH		SYNTENATOR		BF on 3-BRH		EnsEMBL compara	
	mouse	rat	mouse	rat	mouse	rat	mouse	rat	mouse	rat
Number of blocks	1044	1044	933	933	275	275	1337	1337	150	152
Mean length	18.01	17.54	20.41	19.90	78.43	75.70	13.99	13.70	154.51	148.39
Genes in segment	18798	18310	19045	18568	21569	20818	18707	18321	23177	22556
Genes in blocks	14777	14777	15110	15110	21569	20818	15534	15534	n.d.	n.d.
Block size	1770.8 Mb	1870.0 Mb	1791.5 Mb	1891.5 Mb	2087.5 Mb	2217.9 Mb	1618.7 Mb	1705.3 Mb	2431.2 Mb	2600.7 Mb

Method	BF on EnsEMBL orthologs		BF on BRH		SYNTENATOR		BF on 3-BRH		EnsEMBL compara	
	mouse	dog	mouse	dog	mouse	dog	mouse	dog	mouse	dog
Number of blocks	989	989	987	987	458	458	1233	1233	361	355
Mean length	17.60	15.13	17.07	14.63	40.94	34.78	13.40	11.63	61.19	48.07
Genes in segment	17406	14965	16848	14439	18750	15931	16527	14337	22090	17064
Genes in blocks	12840	12840	12595	12595	18750	15931	12836	12836	n.d.	n.d.
Block size	1827.3 Mb	1786.7 Mb	1723.2 Mb	1679.5 Mb	1874.6 Mb	1820.8 Mb	1592.9 Mb	1571.7 Mb	2329.9 Mb	2168.4 Mb

Table 4: **Comparison of conserved synteny predictions for genome pairs**. BF stands for BLOCKFINDER. BRH denotes Best reciprocal BLAST hits. Genome data and compara data are retrieved from the EnsEMBL database release 40.
